# Multiplex quantification of endocrine proteins in volumetric dried blood spots

**DOI:** 10.1186/s12014-025-09539-3

**Published:** 2025-05-09

**Authors:** William Stauch, Johan Olausson, Annika Bendes, Olof Beck, Jochen M. Schwenk

**Affiliations:** 1 Science for Life Laboratory, Department of Protein Science, KTH Royal Institute of Technology, Stockholm, Sweden; 2 Department of Laboratory Medicine, Clinical Chemistry, Östersund Hospital, Östersund, Sweden; 3 Department of Medical Sciences, Clinical Chemistry, Uppsala University, Uppsala, Sweden; 4 Capitainer AB, Solna Torg 19, Solna, Sweden; 5 Department of Clinical Neuroscience, Karolinska Institutet, Stockholm, Sweden

**Keywords:** Dried blood spots, Multiplexed immunoassays, Endocrine hormones, Women’s health, Quantification

## Abstract

**Background:**

Circulating proteins are routinely quantified from liquid biopsies to deduce health and disease. Among these are endocrine protein hormones, which regulate human growth, development, metabolism, and reproduction. Most commonly, these proteins are analyzed in plasma or serum prepared from venous blood draws. Recently, devices for quantitative capillary sampling from a finger prick have emerged, but their utility for clinical testing remains to be explored.

**Methods:**

To study the analytical capabilities of quantitative dried blood spots (qDBS), we quantified the luteinizing hormone subunit beta (LHB), follicle-stimulating hormone subunit beta (FSHB), thyroid-stimulating hormone subunit beta (TSHB), prolactin (PRL), and growth hormone 1 (GH1) by multiplexed immunoassays. We determined the performance of the endocrine hormone assays in paired qDBS and EDTA plasma samples from 100 donors (90% females) aged 4 to 78. Lastly, we compared the protein levels with those from an accredited clinical chemistry laboratory.

**Results:**

The multiplexed analysis showed precise protein quantifications in qDBS (mean CV = 8.3%), high concordance with plasma levels (r = 0.88 to 0.99), and accuracy being matrix- and protein-dependent (recovery: 80–225%). Using the current protocol and sample dilutions, reported protein concentrations were 1.2 to 7.5 times higher in plasma than in qDBS eluates. Concentrations from multiplexed plasma assays agreed with the clinical data (r = 0.87 to 0.99) and decreased slightly when comparing clinical plasma data with multiplexed qDBS assays (r = 0.76 to 0.98). Significant increases in age-related FSHB and LHB levels were observed in females in all specimens and assays (p < 0.01).

**Conclusions:**

This study shows the suitability of modern qDBS devices for quantifying clinically informative proteins in multiplexed assays and highlights the need for future work on specimen-specific optimization and standards. Volumetric DBS sampling offers new routines for accurate protein quantification for precision medicine.

**Supplementary Information:**

The online version contains supplementary material available at 10.1186/s12014-025-09539-3.

## Background

Current routines in laboratory medicine quantify circulating biomarkers in plasma and serum prepared from venous blood draws. However, phlebotomy is a medical procedure that must be performed by trained healthcare professionals and in health facilities [1]. An alternative to clinical specimens obtained from venous blood is dried blood spots (DBS), which can be obtained from capillary blood by finger-pricking. An advantage of DBS is that it does not necessitate the involvement of healthcare personnel, which increases the options for donors to sample themselves at home and can be done at any suitable time [2].

DBS sampling has for over 60 years been used in a clinical setting for newborn screening since Guthrie introduced the screening for Phenylketonuria (PKU) in 1963 [3, 4], an application where exact quantification is not needed. However, traditional DBS struggles with sample volume uncertainty and the hematocrit effect [5]. In such cases, traditional DBS samples introduce variations in blood sample volume, leading to unfavorable measurement uncertainty. To overcome this limitation, volumetric DBS devices allow for exact blood volume sampling [6–8]. Applying accurate volumes of samples to the analytical system is crucial since deviations between the expected and loaded amounts affect clinical decision-making. Previously, the usefulness of volumetric DBS devices has been demonstrated during clinical trials for therapeutic drug monitoring [9]. Additionally, they have been used for home sampling, where the devices were sent to the laboratory for analysis by regular mail [10]. Such an approach was also used during the recent pandemic to understand how SARS-CoV-2 affected the general population [11, 12] .

For molecular analyses, immunoassays are well-established methods that allow measuring the concentrations of single or several proteins in clinical samples at a time [13]. Over the last decades, immuno-based platforms have generally been preferred for clinical diagnosis over chromatographic technologies. They offer robust, simple, automated, and sensitive routines, yielding high-throughput assays suitable for routine measurements in clinical laboratories [14]. One persistent advantage of immunoassays over mass spectrometry (MS) has been their ability to detect circulating proteins at low concentrations (≤ 1 pg/ml, 15).

One of several technologies used to study many proteins in one assay is based on color-coded particles [16]. Multiplexing is achieved by conjugating target-specific antibodies to uniquely color-coded magnetic beads and mixing beads created for different analytes. Parallel detection is then achieved by a fluorescent reporter molecule, and a flow cytometer registers the intensity of the reporter and the bead ID simultaneously. Together with analyzing representative standards, the method has the flexibility to quantify several proteins in one blood sample [17].

This study aimed to test the analysis of dried blood samples for quantifying proteins of clinical relevance, choosing five circulating hormones from the endocrine system [18]. All proteins are secreted from the pituitary gland and affect processes such as development and metabolism, but they have also been used as one cornerstone in assessing female fertility [19, 20]. So far, phlebotomy is a routine procedure for blood sampling, and measured blood levels can be compared to reference values. However, implementing home-sampling devices for capillary blood sampling would offer a more flexible procedure to collect a sample at the right point in time. Self-sampling would also reduce costs and save time [21]. Thus, we identify an increased need for clinical validation of volumetric DBS devices as specimens for protein quantification, which is essential in indications requiring monitoring exact concentrations.

Here, we performed a proof-of-concept study comparing paired plasma and quantitative dried blood spot (qDBS) samples from 100 donors, analyzing five proteins secreted from the endocrine system. The different sample types were investigated in multiplexed assays (Luminex) and compared with data from clinical chemistry (Roche Diagnostics).

Methods

### Samples

In this study, surplus sample volumes from routine clinical hematology testing at the Departments of Clinical Chemistry, Uppsala, were utilized. A set of 100 anonymized samples with known age and sex (90 females and 10 males) were used to evaluate microfluidic cards as a sampling method before clinical chemistry analysis of four different endocrine hormones. The blood was first applied to the microfluidic qDBS cards (*Capitainer®B*), as described below. Subsequently, the tubes were centrifugated for 10 min at 2500 × g at 23 °C and the obtained plasma was transferred to tubes. This study was approved by the ethical committee at Uppsala University (DNR 01-367).

### Materials

Phosphate-buffered saline (PBS) was acquired from Medicargo (#09-9400-100, LOT:244,219) and prepared by dissolving one tablet in 1 L MilliQ water. Tween 20 was acquired from Thermo Fisher (#BP337-500, LOT: 194,435). Complete Mini Protease Inhibitor Cocktail was acquired from Roche (#04693116001, LOT: 45,868,700) and prepared by dissolving one tablet in 2 mL MilliQ water. An elution buffer to extract proteins from qDBS was prepared from PBS with 0.05% tween 20 (PBS-T) and 4% protein inhibitor cocktail.

The 96-well plates hosting the punched qDBS samples and protein extraction were acquired from VWR (734-2781, LOT: 220920078F). The 96-well plates for centrifugation of qDBS extracts, storage, and plasma dilution were acquired from Thermo Scientific (AB-0600). The 96-well plates used for the protein analysis were from Greiner (675,101, LOT: E22103UT). The tubes to collect blood samples were K2-EDTA tubes (364,664, BD Vacutainer Systems Plymouth, UK). Cryo tubes (polypropylene) were used to store plasma.

The multiplex assays targeted the proteins luteinizing hormone subunit beta (LHB), thyroid stimulating hormone subunit beta (TSHB), follicle-stimulating hormone subunit beta (FSHB), growth hormone 1 (GH1), and prolactin (PRL), was from Bio-Rad (#171AHR1CK, LOT: K21748). 

### Sample preparation

*Capitainer*^*®*^*B* is a new generation quantitative dried blood spot (qDBS) microsampling card available for home sampling [22]. This technology is based on a microfluidic technique wherein blood is metered by a 10 µL capillary within the device to deliver an exact volume to a pre-cut DBS disc. This technique minimizes issues with over or under-filling, and differences in volume and analytical distribution due to varying hematocrit, seen in conventional DBS, and allows for quantitative measurements in downstream analysis [7, 23]. Each card collects samples in two separate paths (two sample discs). In this study, EDTA blood collection tubes were mixed to resuspend the blood cells, and then, 25 µL of the whole blood solutions were added to each of the two sample wells on the collection cards. After approximately 10 s, the metering process was completed, indicated by a red dot that appeared above the sample and a visual indicator for successful sampling. Before analysis, all samples were retrieved by opening the protective tabs on the back of the cards, and the dried sample discs were transferred to 96-well plates. To extract the proteins from the discs, 100 µL of elution buffer was added to each well and incubated for 60 min at 23 °C during shaking. The extracts were used for downstream analysis, and the results were compared to the plasma measurements conducted on cobas pro (Roche Diagnostics).

### Sample storage

The plasma samples for the clinical chemistry analysis were stored at -20 °C for 10 weeks before analysis on Cobas Pro. A second aliquot of plasma samples was delivered fresh with corresponding qDBS cards for analysis using multiplex immunoassays. These plasma samples were stored at − 80 °C for 10 weeks, while the qDBS cards were stored at 23 °C for 4 weeks until extraction.

### qDBS elution

Barcoded qDBS cards were prepared for downstream elution by a fully automatic card handler (PA496, Capitainer AB). Firstly, barcoded cards were loaded onto racks and barcoded 96-well plates, scanned and registered in their position. A gripper arm picks each card, registers the barcode of the card, and a built-in camera captures the status of the sample discs. If the sample disc was registered as correctly filled, it ejects the pre-cut filter-paper disc into a specific position of a 96-well plate. Again, a camera captures an image of the disc in the well. This ensures that the correct disc is placed in the designated position along with documentation for traceability. The fully automated system allows laboratory staff to perform other tasks while the instrument is in operation.

Protein extraction was performed by adding 100 µL elution buffer to each of the wells of the plates. Therefore, the qDBS samples were diluted 1:10, since 10 µl dried blood was eluted in 100 µl elution buffer. The discs were then incubated in an elution buffer with gentle agitation for 1 h, 170 rpm at 23 °C. These eluates were transferred with a manual multichannel pipette to fresh 96-well plates. Subsequently, the plates were centrifuged at 3000 rpm for 3 min (Allegra V-15R, Beckman Coulter) to sediment insoluble material, and 65 µl of the supernatant was thereafter transferred to fresh plates and at − 80 °C for 6 weeks.

### Multiplexed assays

The multiplex assays were conducted according to the manufacturer’s instructions. Plasma samples and qDBS extracts were thawed at 4 °C for 1 h and vortexed and centrifuged for 15 min, 1000 × g, 4 °C. The plasma samples were diluted (1:5) with sample diluent, supplied in the kit, to 100 µL in a 96-well plate, while qDBS eluates were used undiluted. Paired plasma and qDBS samples from 100 donors were distributed in random positions of three 96-well plates (paired samples were kept on the same plates) and analyzed on two consecutive days. As instructed by the kit, two controls, blank samples, and an eight-point standard curve were measured in duplicates for each plate.

Blocking buffer (10 µL), sample (30 µL), and capture beads (10 µL) were added to a 96-well plate. The plate was incubated on a plate shaker, 850 rpm, for 1 h at 23 °C, followed by washing (3 × 100 µL, kit-specific wash buffer) using an automated plate washer (BioTek, EL406). A secondary antibody (40 µL) was added, followed by an additional 1 h incubation with agitation (same setting as above). Streptavidin-coupled fluorophore (20 µL) was then added for 30 min incubation on the plate shaker (same setting as above). Subsequently, the plate was washed (3 × 100 µL) with an automated plate washer, and 100 µL wash buffer was dispensed to the wells. Lastly, the plate was incubated for 30 s on the plate shaker (same setting as above) before analysis with Luminex MagPix (xPONENT 4.2.1705.0).

### Calibration curve

The calibration curve was repeated for each plate and measured in duplicate. The calibrator provided with the kits was designed for plasma and serum samples. In this study, it was also applied to quantify proteins in qDBS. A dose–response curve was generated from the proportional relationship between the recorded intensity and the analyte concentration. A five-parameter logistic function estimated a curve model (Eq. 1), and the protein concentration could be calculated by the inverse of the function (Eq. 2). Protein concentrations were corrected for dilution factor: 1:5 for plasma samples and 1:10 for the qDBS samples

1 $$y(\text{x})=c+\frac{(d-c)}{{(1+{\left(\frac{x}{e}\right)}^{b})}^{f}}$$

2 $$x=e{({\left(\frac{d-c}{y-c}\right)}^\frac{1}{f}-1)}^\frac{1}{b}$$

### Imprecision

To determine the inter- and intra-assay precision, replicates of two sets of pooled qDBS samples were used to measure the inter- and intra-plate coefficient of variation (CV). The CVs were based on median fluorescence intensity (MFI) reported by the Luminex system. In two of the plates, the pools were measured in quadruplicates, and in the third plate, the two pools were measured in triplicates.

### Matrix effect

Dried whole blood and plasma have a different protein composition; thus, different background effects may occur. Subsequently, the matrix effect was evaluated by a spike-in, and its recovery was observed. Three qDBS pools and plasma pools were spiked with a diluted calibrator. Three different dilutions of calibrator were used as spike and added to each pool: dilution factors 1:20, 1:60 and 1:180. The spiked pools were then compared to neat, pooled samples, which had an equivalent volume of added PBS.

The preparation of the spikes was prepared accordingly: spike with a final dilution of 1:20 consisted of undiluted calibrator; the 1:60 diluted spike was prepared by firstly diluting the calibrator (1:3) in standard diluent; the 1:180 diluted calibrator was prepared by firstly diluting the calibrator (1:9) in standard diluent. The prepared spike (5 µL) was then added to (95 µL) pooled qDBS extract, or pooled plasma (diluted 1:5 in sample diluent, priorly). Consequently, yielding the three final dilutions of the calibrators: 1:20, 1:60 and 1:180. The measurement was conducted in triplicates and reported as the average of these. The theoretical protein concentrations of the spiked proteins were compared to the difference between the recovered concentration from the spiked and neat samples, providing the assay recovery (**Eq. **3). Subsequently, the average recovery of the three spikes (1:20, 1:60 and 1:180) was reported.

3 $$Recovery= \frac{(measured conc. spiked sample - measured conc. neat sample)}{conc. of the spike}$$

### Clinical reference method

The sex hormones FSHB, LHB, PRL and TSHB were analyzed in plasma using cobas pro e801 (Roche Diagnostics, Rotkreuz, Switzerland). The cobas pro employs electrochemiluminescence for all four assays (FSHB #08932387190, LHB #07027575190, PRL II #07027737190, TSHB #08443432190). Measurements on cobas pro was conducted at the Department of Clinical Chemistry, Östersund Hospital. The laboratory and the assays participate in the external quality assurance program run by Equalis (Uppsala, Sweden). Results from cobas pro were considered reference values in this study. The detection metrics for the cobas pro are displayed in Table S1.

### Data processing and analysis

Data analysis and visualizations were performed in R statistical software (v4.3.2). The standard curves were generated by applying a five-parameter log-logistic curve to the data points using the *drc* package [24]. The sample concentration was interpolated from the standard curve. PRL units from the clinical chemistry analysis were converted from µIU/mL to ng/mL by the conversion factor from the current International Standard for PRL [25]. The lower limit of detection (LOD) and the lower limit of quantification (LOQ) were determined as 3 × SD and 10 × SD over the mean of the blank. Samples with signals below LOD and above the top standard point were excluded from further analysis for the specific target. Metrics for Pearson correlation and regression analyses were computed with *stat_cor()* and *lm()* functions.

## Results

**Fig. 1:**
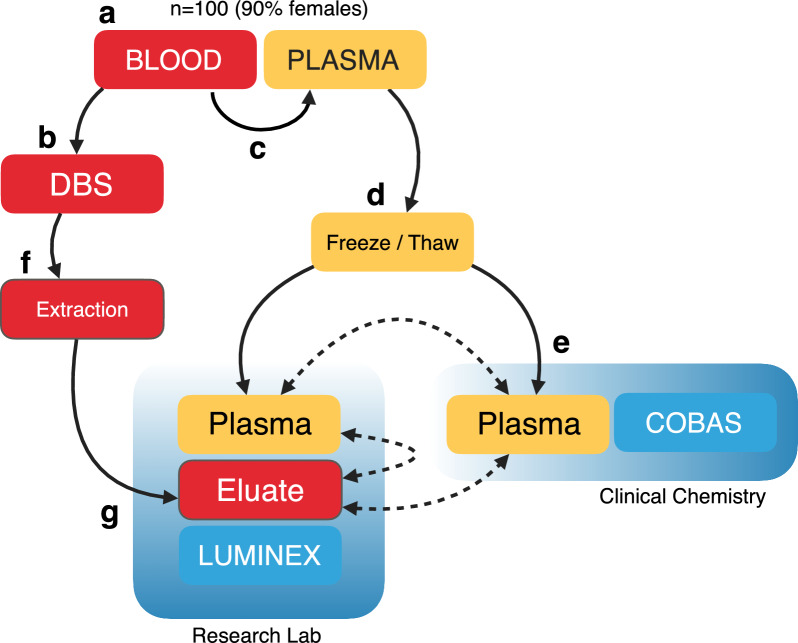
Study design—**a** Venous blood samples were collected from 100 participants in EDTA tubes as part of clinical routine procedures. **b** Whole blood was transferred onto qDBS cards and let to dry. **c** Plasma was collected after centrifugation from the supernatant and **d** frozen until it was thawed for **e** the analysis in the routine flow of the clinical chemistry lab (cobas pro). **f** The dried qDBS discs were punched out and incubated with a detergent-containing buffer to extract the proteins. **g** The eluates of qDBS and paired plasma were analyzed using multiplexed immunoassays (Luminex). The solid lines indicate sample processing steps, and the dashed lines are comparative data analyses

Our study aimed to investigate the performance of detecting proteins secreted by the pituitary gland in dried blood spot samples by using multiplexed immunoassays. As illustrated in Fig. 1, we used donors’ whole blood and EDTA plasma to prepare the samples for protein analyses. Whole blood was transferred onto collection cards to create qDBS and left to dry. Subsequently, the qDBS filter papers discs were incubated with a detergent-containing buffer to elute the proteins. Frozen plasma samples were thawed and analyzed alongside eluted qDBS samples by research-grade multiplexed immunoassays. In parallel, plasma samples were measured by standard protocols in an accredited clinical chemistry lab.

### Analytical performance of multiplexed immunoassays

In the following, we studied several analytical quality criteria (imprecision, recovery, matrix effects, CVs) and compared protein quantities in plasma, qDBS, and the analytical platforms. For the analysis, we chose a commercially available multiplexed immunoassay kit based on bead-based technology (Luminex).

### Comparison of qDBS vs plasma

**Fig. 2:**
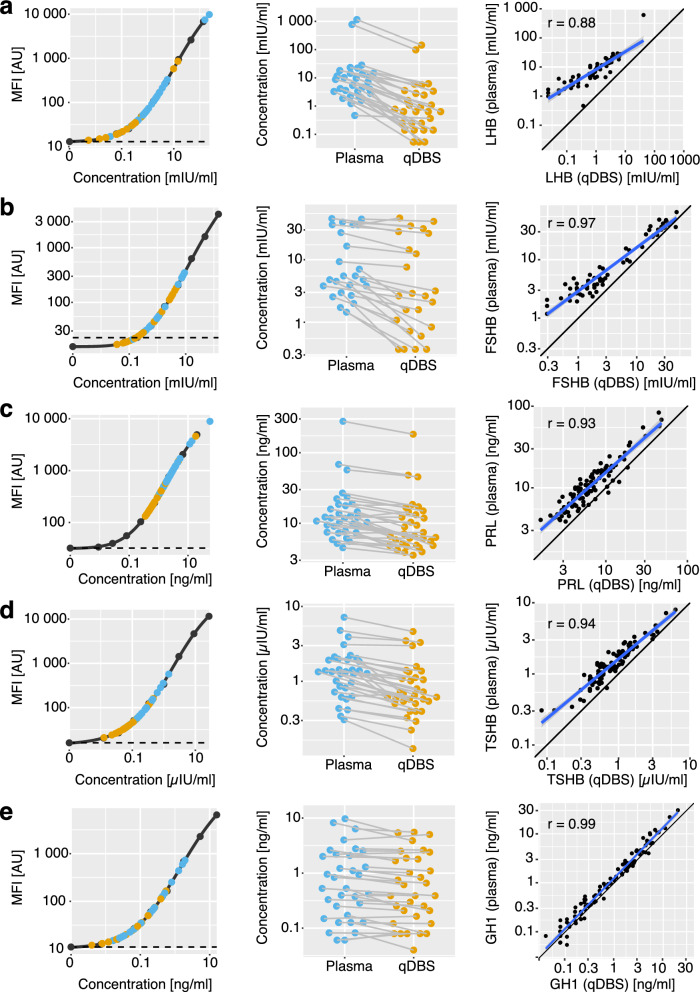
Comparison of protein levels in EDTA plasma and qDBS. Five proteins were measured in one multiplexed immunoassay (Luminex) to compare the concentrations between eluates from qDBS and EDTA plasma samples: **a** LHB; **b** FSHB; **c** PRL; **d** TSHB; **e** GH1. Left column: Dose–response curve of plate 1 with a subset of subjects (n = 33); qDBS samples are depicted in yellow, plasma in light blue, and values from the calibration curve in black. The limit of quantification (LOQ) is shown by a black dashed line. Center column: protein concentrations in paired qDBS and plasma samples are adjusted for the preparatory dilution factors. Right column: The correlation between plasma and qDBS for all subjects (n = 100) above LOQ and below the highest point on the calibration curve. The blue line shows the linear regression model, and the 95% confidence intervals are in grey. Pearson r demonstrates the correlation, and the identity line is shown in black

First, we created standard curves for each hormone analyte and different assay plates; see the first column in Fig. 2 for plate 1 (Fig. S1-2 for plates 2 and 3). The response curves were similar between plates; the average CV for the standard curve across all plates and targets was 6% (Fig. S3). Projecting the measured values for plasma and qDBS onto these curves, we found inter-individual and inter-specimen differences in concentrations. Individuals with elevated or reduced levels are identified in both sample types and, to a noticeable extent, the ranking of the samples agreed between the specimen (Fig. 2). Plasma revealed higher concentrations than qDBS eluates. We attributed this to qDBS eluates being a more diluted sample matrix than cell-free plasma, referring to the overall lower total protein concentration despite the presence of proteins form blood cells. We also note that the standards were optimized for plasma or serum and not DBS. Despite compensating for the abovementioned differences in dilution, borrowing a standard from a related sample type might have further affected the determined protein levels. This preparatory dilution caused more protein in qDBS samples to be below LOD than in plasma (33 and 32 samples for LHB and FSHB, respectively). LHB and FSHB were below LOD in only 3 of 100 plasma samples. PRL, GH1, and TSHB were detected in all samples. In contrast, nine (FSHB) and two (LHB) samples were detected above the highest point on the standard in plasma. Whereas for qDBS, no samples were detected above the highest point on the standard curve. Overall, the correlation between the two sample types was high for all the proteins (r = 0.88–0.99), as shown in the last column of Fig. 2. Protein concentrations were slightly higher in plasma for all analytes but GH1. The steepness of the regression lines showed differences in the analytical resolution between the two sample types, with GH1 assays performing equally and LHB agreeing to the least. The metrics of the comparative sample analysis of the five proteins can be found in Table S2.

### Evaluation of assay precision and sensitivity

The assay precision was evaluated by two qDBS pools. Each pool was measured with quadruplicated replicates in the first two plates and triplicated replicates in the third plate. Altogether, the evaluation was based on 22 measurements. The intra-plate CVs were all below 8% and close to the precision reported by the supplier (Table 1A). Although the CV was higher in LHB, 7.1%, than the reported CV from the supplier, 4%. While for PRL, the CV was lower, 5.39%, than the CV reported by the supplier, 7%. The inter-plate CV was below 10% for all hormones except for PRL, which had an elevated CV of 16.5%. This was due to one of the plates having an elevated response for PRL, which was displayed by the kit-specific controls (Fig. S4-5). Excluding PRL, the inter-plate CV was below 10% and only moderately deviating from the precision reported by the manufacturer.

**Table 1:** Precision of multiplex immunoassays

%CVs	LHB	FSHB	PRL	TSHB	GH1
Intra-plate DBS (lab)	7.1	6.5	5.4	4.8	7.1
Intra-plate plasma (supplier)	4	7	7	5	6
Inter-plate DBS (lab)	9.8	8.1	16.5	8.5	9.5
Inter-plate plasma (supplier)	6	8	8	7	7

Coefficient of variation (%CV) as specified by the supplier and our replicated analyses (n = 22) based on MFI. Intra*-*plate CVs from qDBS were calculated from the mean of six independent samples. The inter*-*plate CVs from qDBS samples were based on three assay plates.

Furthermore, the determined limits of detection (LOD) in were consistently close to the LOD reported by the manufacturer (Table S3). Again, PRL had a higher LOD, 0.013 ng/ml, than the LOD specified by the supplier, 0.0077 ng/ml. Likewise, did the multiplexed TSHB assay have an LOD higher than specified by the manufacturer, 0.0080 µIU/ml versus 0.0044 µIU/ml. For LHB, the concentration of the LOD was only available for one plate since the other plates had a concentration lower than the minimum range of the five-parameter function used to interpolate the sample concentrations.

These investigations confirmed the suitability of the data for further analysis, as CVs and LODs were in the range of the supplier-provided performance values.

### Matrix effect

The recovery of spiked plasma and qDBS samples was measured in three pools each. Each pool was spiked with three concentrations of the kit calibrators, containing all five proteins at the defined level, and measured in triplicates. One replicate of a qDBS pool was excluded as it was considered an outlier. The overall average recoveries ranged 80–225%, as shown in Fig. S6. The recovery levels for FSHB and TSHB in qDBS were consistently ≥ 100%; the mean recovery was 122% and 144%, respectively. Their mean recovery in plasma was 93% and 96%, respectively. LHB, on the other hand, showed a near-perfect recovery in plasma of 99%, and in qDBS, a lower mean recovery of 80%. For GH1 and PRL, the recovery was inconsistent using the more diluted calibrator, especially for PRL, yielding high standard deviations. This indicates that the sensitivity and resolution for these two assays were insufficient and would require a greater difference between spiked proteins and neat samples to produce more consistent results.

### Towards matrix-specific thresholds

**Fig. 3:**
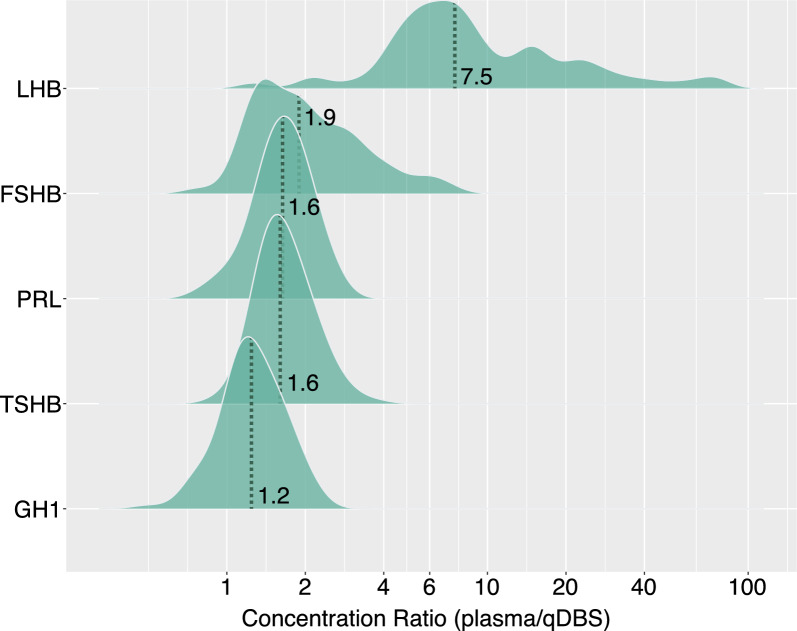
Ratio of plasma and qDBS concentrations. Distribution of concentration ratios (plasma/qDBS) for the five proteins. The median ratio of each hormone is displayed, and its position is marked with dotted vertical lines

As observed above, protein levels in plasma specimens were generally higher than in qDBS, since the assay was developed for plasma and DBS was a more diluted specimen. In Fig. 3, we determined the distributions of concentration ratios between plasma and qDBS in multiplex assays to better judge each protein assay. The ratios were normally distributed for GH1, TSHB, and PRL with median ratios between 1.2 and 1.6. FSHB showed some tailing and an average ratio of 1.9. These four examples reflect a protein assay specific influence of the preparatory dilution effects on DBS, a contribution of the protein composition (matrix) due to the presence of blood cell proteins in DBS, and the use of standards developed for plasma but not specifically for DBS analysis. In comparison, LHB had a broad and inconsistent ratio distribution with a median ratio of 7.5. This was, however, consistent with the decreased sensitivity in qDBS, as shown in the last column of Fig. 2.

To further illustrate how the concentration levels influenced the comparative performance, we prepared Bland–Altman plots in Fig. S7. Reassuringly and consistent with previous data visualizations, there was a good agreement between qDBS and plasma levels because the majority of protein concentrations were within the 95% confidence intervals. Despite differences in sample dilution, sensitivity, resolution, and matrix effects, the variance of protein levels measured in the sample set was in good agreement for both specimens.

### Comparison with clinical data

To independently evaluate the analytical difference of the multiplexed immunoassays, we compared the levels to plasma data from a clinical chemistry laboratory. Studying four hormones (FSHB, LHB, PRL, and TSHB) in two different labs, we first investigated the correlation between the two methods for plasma.

**Fig. 4:**
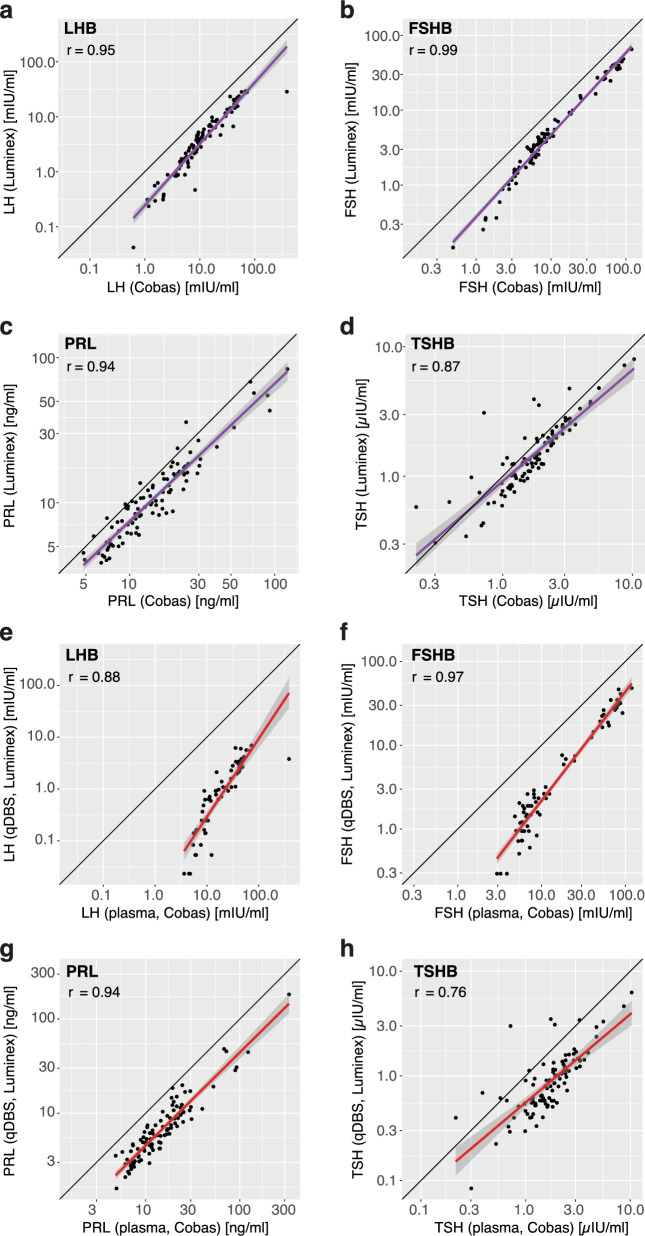
Comparison of EDTA plasma and qDBS concentrations with clinical data. Correlation plot depicting the quantified proteins **a** LHB, **b** FSHB, **c** PRL, and **d** TSHB in EDTA plasma from clinical chemistry analysis (Cobas pro e801) and multiplexed immunoassays (Luminex) from all subjects (n = 100). In the plot **e** LHB, **f** FSHB, **g** PRL, and **H** TSHB, the qDBS data (Luminex) was compared with clinical chemistry analysis. Linear regression lines are shown in purple for plasma vs plasma and red for qDBS versus plasma. The 95% confidence intervals are shown in grey around a linear regression fit in purple or red. The line of identity is shown in black

As shown in Fig. 4A–D, there was a high correlation (r = 0.87 to 0.99) between the independent measurements. The slopes were nearly parallel to the line of identity (b = 0.86 to 1.1), suggesting an on-par performance of the assays. Levels determined by the Cobas analysis were slightly higher than those from Luminex measurement, which can be attributed to using different calibrators. The multiplexed assay revealed higher TSHB levels in a subset of the samples, which might be due to measuring this protein via different epitopes or due to interferences in these samples. The metrics of the comparative plasma analysis can be found in Table S4.

Changing the comparison to qDBS for the multiplex assays retained a high correlation for FSHB, LHB, and PRL (r = 0.94 to 0.97), while the TSHB correlation became lower (r = 0.76), as shown in Fig. 4E–H. Notably, the clinical levels remained higher, but slopes became steeper than in the plasma comparison (b = 0.84 to 1.5). Despite the effect assigned to the qDBS matrix, and higher sample dilution of qDBS and standards developed for plasma the consistency in ranking samples based on their concentration supports the utility of qDBS as a sample type for measuring these hormones. The metrics from the comparative sample type and assay analysis can be found in Table S5.

### Effects of age on protein levels

Age is a well-established factor influencing protein levels in the circulation [26], and for women, hormone levels change with the onset of menopause [27]. To investigate if the qDBS sample collection available from this pilot study can reveal indications for this female biology, we used age to categorize the samples from female donors into pre-menopause (< 45 years), perimenopause (45–55 years), and post-menopause (> 55 years). No other data or clinical information about the donors was available and considered.

**Fig. 5:**
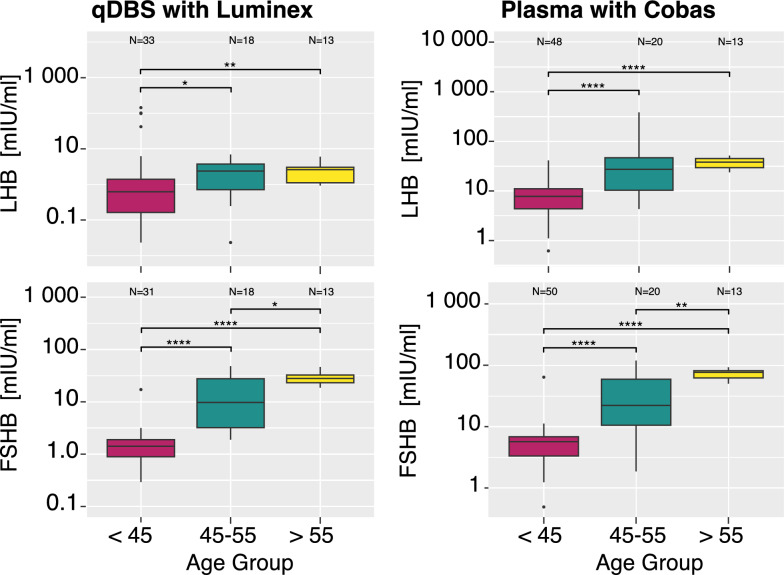
Agee association of protein levels. The boxplots include three age groups of women: < 45 years of age, 45–55, and > 55 years of age for LHB (top row) and FSHB (bottom row). The left plots show qDBS from multiplex assays, while the right plots show plasma analyzed at the clinical chemistry laboratory. The number of subjects with protein levels above LOD is shown above the respective box. Nominal p-values were calculated using Wilcoxon tests and displayed as * p < 0.05; ** p < 0.01; *** p < 0.001; **** p < 0.0001

Among the five tested proteins, changes in FSHB and LHB levels were significantly different (p < 0.05) in the three age groups, regardless of specimen and laboratory technique, as shown in Table 2 and Fig. 5 (and Fig. S8). Differences in the degree of association for LHB were influenced by the number of samples remaining after removing those with concentrations below LOD (n = 33 for qDBS versus n = 48 for Cobas). The results from group-wise comparison and linear regression are shown in Table S6 and Table S7.

**Table 2:** Age group comparison

Sample (platform)	Comparison	LHB	FSHB	PRL	TSHB	GH1
Plasma (Luminex)	Pre-Peri	2 × 10^–4^	3 × 10^–6^	0.5	0.4	0.6
	Peri-Post	0.4	0.02	0.8	0.1	0.4
	Pre-Post	4 × 10^–6^	6 × 10^–8^	0.4	0.4	0.3
qDBS (Luminex)	Pre-Peri	0.03	7 × 10^–7^	0.9	0.7	0.9
	Peri-Post	0.7	0.01	1	0.05	0.7
	Pre-Post	0.003	2 × 10^–7^	1	0.1	0.5
Plasma (Cobas)	Pre-Peri	3 × 10^–5^	4 × 10^–6^	0.4	0.08	N/A
	Peri-Post	0.4	0.006	0.7	0.2	N/A
	Pre-Post	2 × 10^–7^	5 × 10^–8^	0.7	0.7	N/A

For descriptive purposes, we used general information about age and menopause to assign the samples into groups representing pre- (< 45 years), peri- (45–55 years), and post-menopausal (> 55 years) phenotypes in women. Wilcoxon tests were used to determine nominal p-values for the differences in protein concentrations across the age groups in the three data sets

## Discussion

In this study, we used paired plasma and qDBS samples to demonstrate the concordance and difference between dried-up and frozen specimens for measuring clinically relevant proteins. Five clinically relevant hormones were quantified using a multiplex immunoassay with excellent precision and accuracy without major modifications to the assay protocol.

All protein hormones studied here are known to be secreted into the circulation by the pituitary glands [28], see Fig. S9. Since only neglectable RNA expression was found in hematopoietic cells, leakage from blood cells was not expected. Hence, it was reasonable to assume that these circulating hormones can only be found in extracellular fluid and originate from the secreting tissue. Still, the measured concentrations in the plasma samples were always higher than in qDBS. We related this to the need to dilute DBS during the protein extraction process, the use of standards developed for plasma samples, and the differences in the protein composition attributed to the presences of blood cell protein (e.g. hemoglobin) in DBS compared to cell-free plasma.

Approximately 55% of whole blood consists of extracellular fluid [29]; red blood cells predominantly take the remaining volume. In 1 µl of whole blood, there are about 4–6 million red blood cells (RBC), 5–10 thousand blood cells, and 150–400 thousand platelets. The RBC concentration, often reported as hematocrit levels (% cell volume), may vary between individuals and range between 39 and 50% in men and 35–40% in women [30]. Consequently, qDBS is expected to have about 55% of the concentration of the corresponding plasma sample. Hence, we can expect that plasma samples will have a 1.8-times higher concentration than in qDBS. In this study, the ratios between plasma and qDBS for FSHB, TSHB, and PRL were near this range (median was 1.2–1.9). The effect of the sample matrix could explain why these proteins had slightly lower ratios.

In the recovery experiments, a higher response in qDBS than plasma was demonstrated for three proteins: FSHB, TSHB, and GH1. PRL showed inconsistent recovery levels when using the more diluted spike due to lower sensitivity. On the other hand, LHB had a higher ratio than the other proteins and decreased recovery levels in qDBS compared to plasma. This suggests the DBS matrix interfered with this assay, causing greater qDBS and plasma concentration dissimilarities for LHB. This showcases that matrix effects, possibly caused by high concentrations of RBC-derived proteins, can influence protein quantifications and that DBS-derived concentrations are not equivalent to those in plasma. For that reason, new reference concentrations of DBS tests and adapted calibrators for DBS matrices would be needed.

For four of the proteins, the measured concentrations from the multiplex assays could also be compared to those from the routine clinical chemistry measurements in plasma. The two independent measurements and methods were highly correlated when using the same specimen (plasma). When switching the specimen to qDBS, the correlation to the reference measurements was still high for GH1, FSHB, and LHB. TSHB, however, had the poorest correlation between the clinical analysis with plasma and the multiplex assays using qDBS. Since the epitopes for the antibodies used in the two assays are not specified, we can only assume that binding interference caused the observed deviation. It has been reported that autoantibodies could interfere with detecting TSHB in immunoassays in some patients [31].

Testing the qDBS with multiplex assays to analyze clinically relevant targets from the endocrine system shows promising results for accurate measurements of circulating hormones. In our study, we were guided by the fact that female fertility ends at menopause, generally occurring in women aged 45–55 years. Earlier studies have shown that FSHB and LHB levels increase in women with age and are associated with different fertility stages [27]. We could confirm that FSHB and LHB levels significantly increased with age, and such differences were observed in both specimen and assay platforms. This supports the feasibility and reliability of qDBS as an alternative sampling method for routine quantification of fertility-related proteins in a clinical setting.

Our study also has limitations. One limitation was that we did not use finger-pricking to load the qDBS cards with capillary blood. Instead, the qDBS cards were filled with leftover EDTA-treated blood from a venous puncture to investigate the effect of analyzing dried-up and resolubilized proteins. We also focused on secreted proteins originating from a single organ and have not studied proteins that can be expressed by other tissues or found in hematopoietic cells. Another limitation is that we only used antibody-based assays to quantify the proteins, and future investigations could include targeted mass spectrometry. Nonetheless, we confirmed our observations by independent measurements and were able to time the measurement to the same calendar week to avoid the influence of storage time.

Overall, the analysis of dried proteins in qDBS has the potential to become an alternative to venous blood sampling, which requires immediate processing, cooling, or freezing of a sample. The option of drying a sample as qDBS without the need to process fluids in a specific and time-sensitive manner could further simplify clinical routines for collecting samples for diagnostic or research purposes. Such an advantage adds to the ease of use for home sampling of capillary blood by finger-pricking. This has the potential for time and cost-savings in the medical field and health monitoring, but the uptake of qDBS in routine laboratory medicine is still slow.

## Conclusions

Using multiplexed immunoassays, we demonstrate that clinically relevant proteins can be accurately and precisely quantified in 10 µL of dried blood. The correlation between results obtained from eluates of qDBS, EDTA plasma, and data from clinical chemistry supports the usefulness of qDBS-based approaches for clinical routine laboratory medicine.

## Supplementary Information

Supplementary material 1.

## Data Availability

The data can be made available for non-commercial validation purposes. A reasonable request should contain (i) The scientific purpose of the data access request, (ii) a Commitment to inform when the data has been used in a publication, (iii) a Commitment not to host or forward share the data outside the requesting legal entity, and (iv) a Statement of non-commercial use of data.

## Abbreviations

DBS: Dried blood spot
qDBS: Quantitative dried blood spot
PBS: Phosphate-buffered saline
TSHB: Thyroid-stimulating hormone beta subunit
FSHB: Follicle-stimulating hormone beta subunit
GH1: Growth hormone 1
PRL: Prolactin
LHB: Luteinizing hormone beta subunit
RBC: Red blood cells
