# Supplementary material for: Multiplex quantification of endocrine proteins in volumetric dried blood spots
Source: Clin Proteomics. 2025 May 9;22:18. doi: 10.1186/s12014-025-09539-3 (PMC12063380; doi:10.1186/s12014-025-09539-3)
Supplement: Supplementary file 1 — Supplementary material 1. [file 12014_2025_9539_MOESM1_ESM.pdf]

Supplementary figures and tables for

Multiplex quantification of endocrine proteins in volumetric dried blood spot specimens

William Stauch[1], Johan Olausson[2,3,4], Annika Bendes[1],
Olof Beck[5,#,*], and Jochen M Schwenk[1,#,*]

Table of contents:

Supplementary Figs 1-6
Supplementary Table 1

Figure S1. Calibration curve for plate 2 and the concentration of the samples - Dose-response curve and concentration of paired qDBS and plasma samples (n = 33 subjects). plasma samples are depicted in light blue, qDBS in yellow, and values from the calibration curve in black. The limit of quantification is shown by a black dashed line. (a) luteinizing hormone (LHB); (b) follicle-stimulating hormone (FSHB); C) prolactin (PRL); (d) thyroid stimulating hormone (TSHB); (e) growth hormone (GH1).

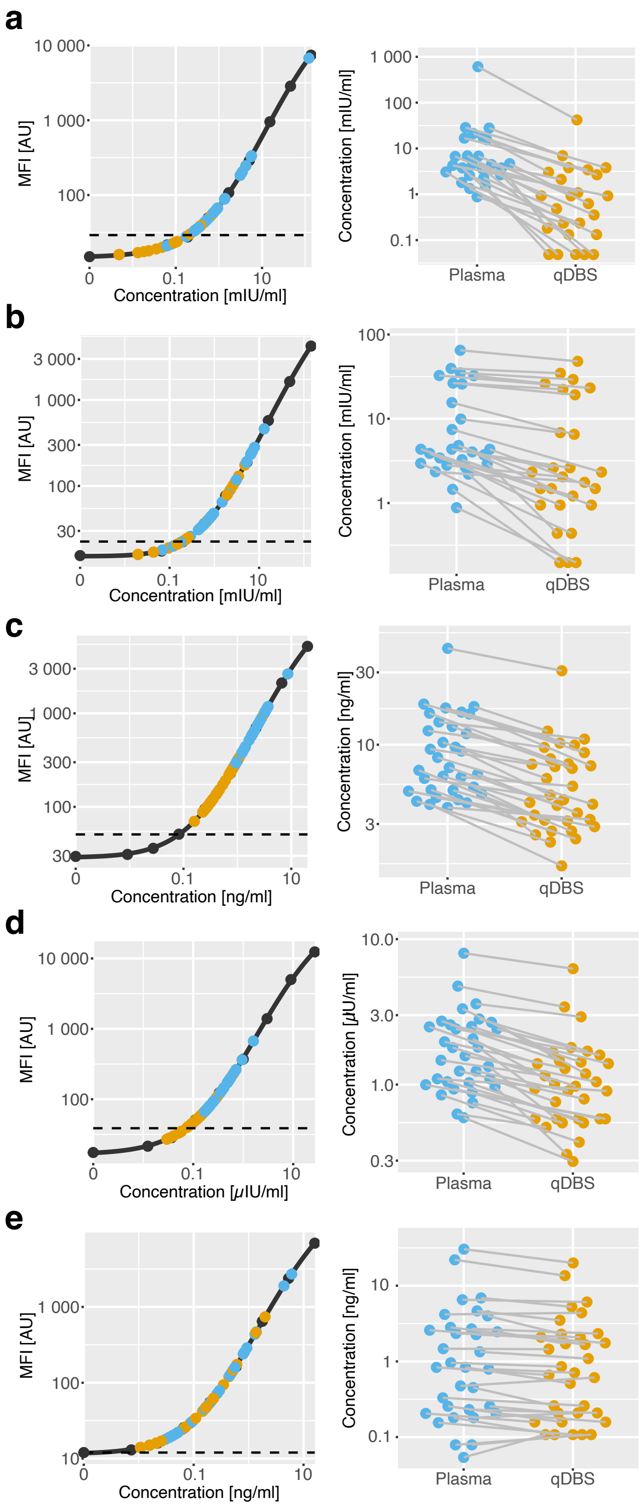

Figure S2. Calibration curve for plate 3 and the concentration of the samples - Dose-response curve and concentration of paired qDBS and plasma samples (n = 34 subjects. plasma samples are depicted in light blue, qDBS in yellow, and values from the calibration curve in black. The limit of quantification is shown by a black dashed line. (a) LHB; (b) FSHB; (c) PRL; (d) TSHB; (e) GH1.

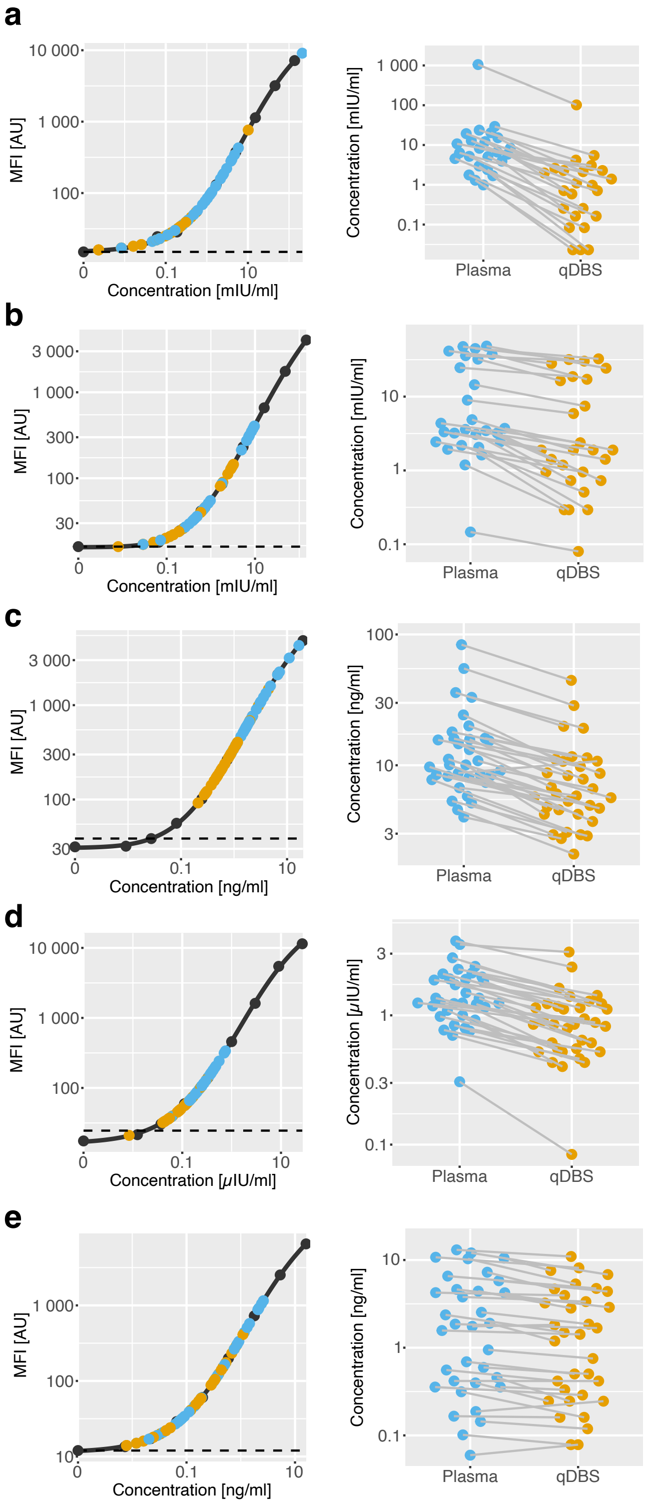

Figure S3. Dose-response curves. Three curves from the calibration are presented. Plate 1 is depicted in yellow, plate 2 is colored blue and plate 3 is orange. (a) LHB; (b) FSHB; (c) PRL; (d) TSHB; (e) GH1.

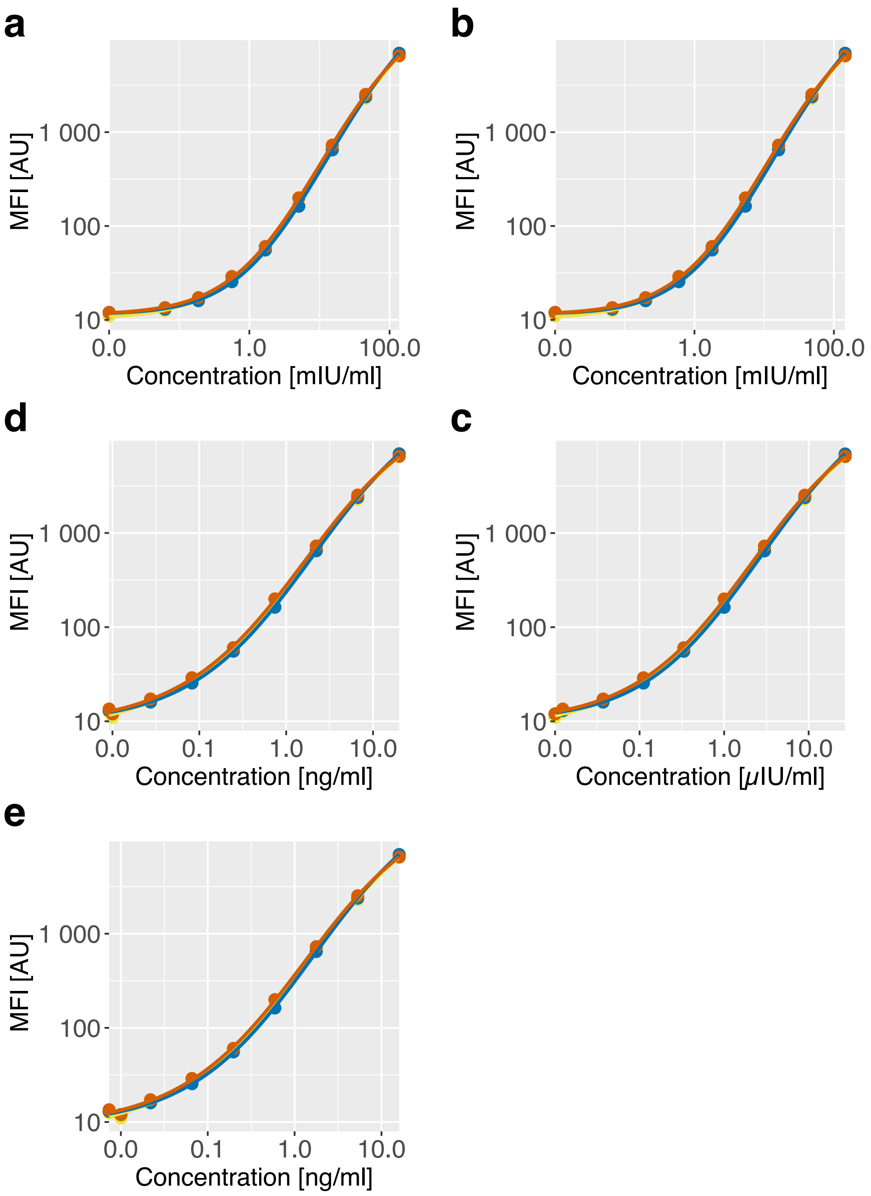

Figure S4. Kit-specific controls. Concentrations for the high kit-specific control, measured with duplicates, and the expected concentration range of the control (dashed lines) for (a) LHB; (b) FSHB; (c) PRL; (d) TSHB; and (e) GH1.

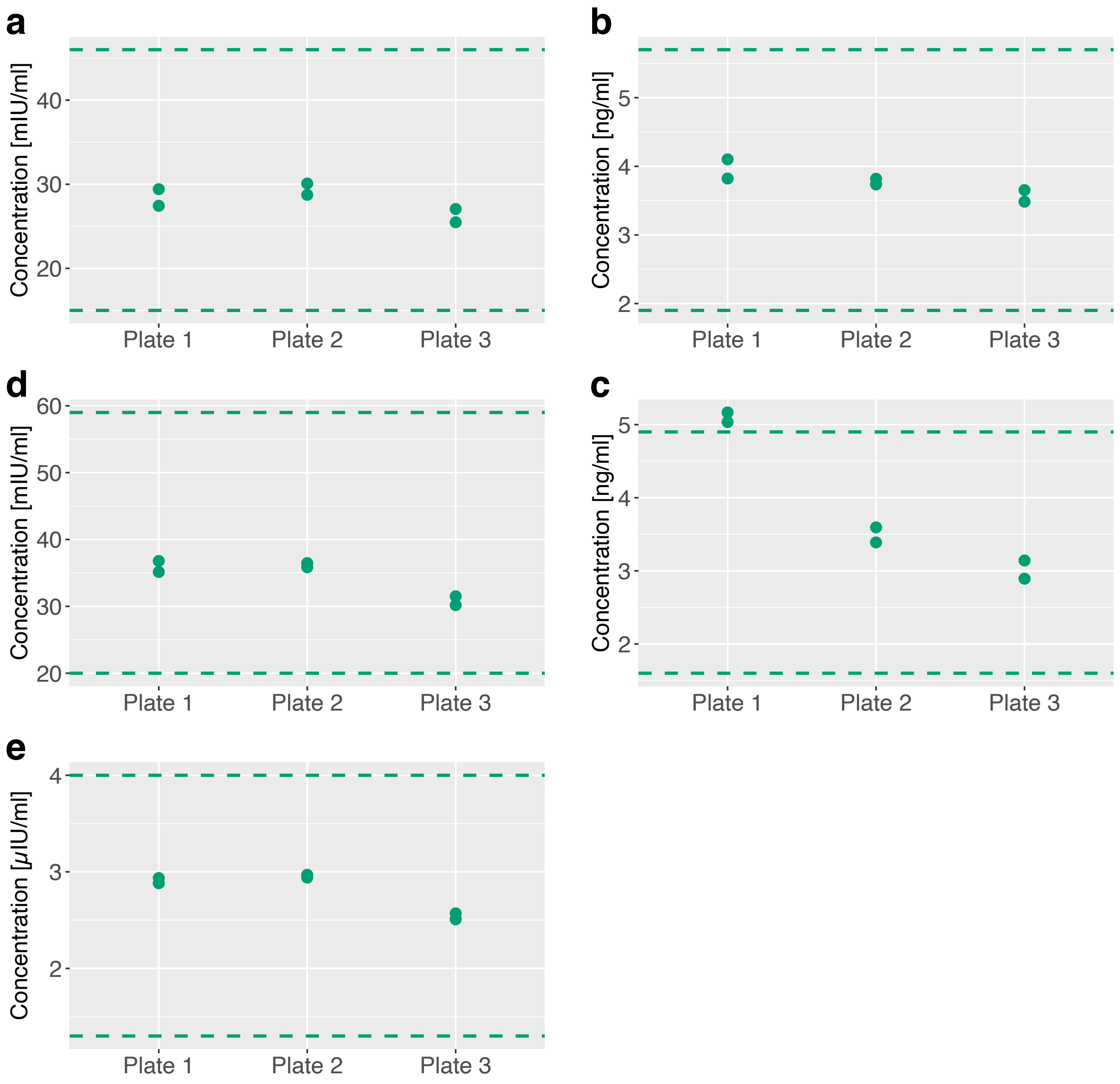

Figure S5. Kit-specific controls. Concentrations for the low kit-specific control, measured with duplicates, and the expected concentration range of the control (dashed lines) for (a) LHB; (b) FSHB; (c) PRL; (d) TSHB; and (e) GH1.

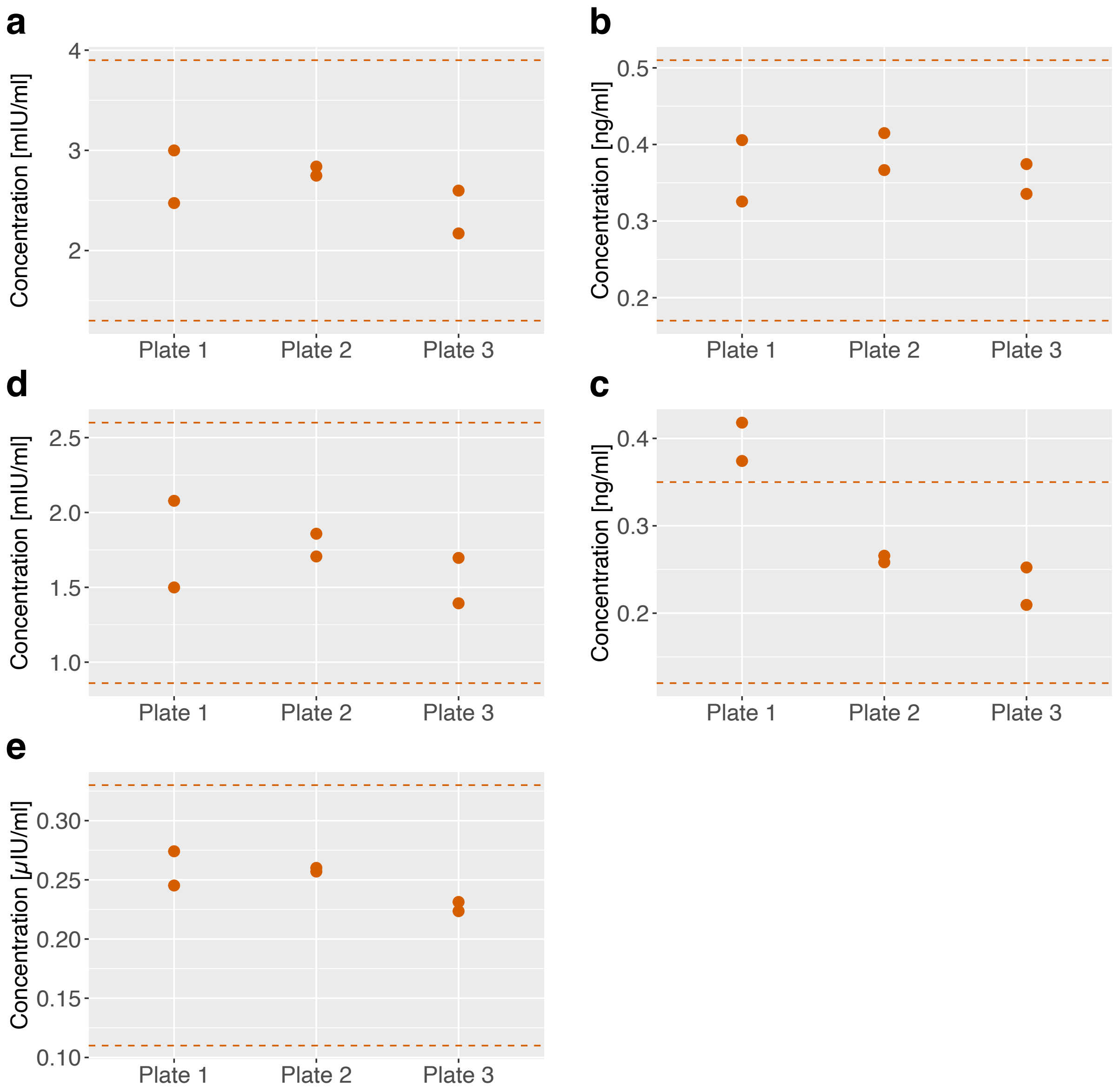

Figure S6. Recovery from spiked qDBS and plasma. Bar plot showing the mean recovery. Three pools of plasma and qDBS were each spiked with three concentrations of diluted calibrator and measured in triplicate. Plasma is depicted in blue and qDBS in yellow. Error bars show the standard deviation of the measurements.

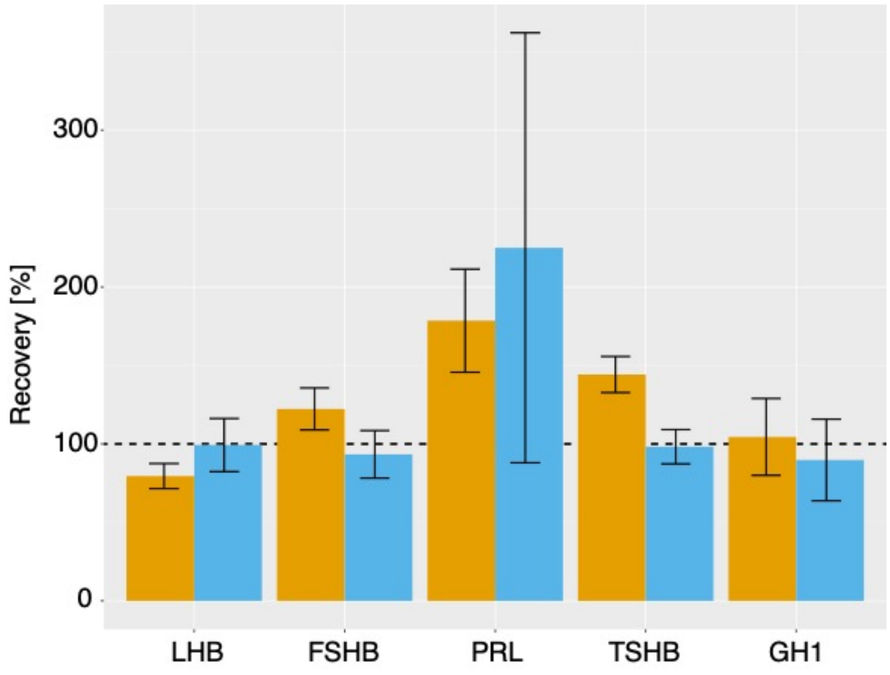

Figure S7. Bland-Altman plot comparing plasma and qDBS. The ratio (qDBS/plasma) is plotted against the average concentration (in log2) of qDBS and plasma. Levels were determined with multiplex immunoassays. The average concentration ratio is displayed as a black dashed line, while the red dashed lines show the 95% confidence intervals. (a) LHB; (b) FSHB; (c) PRL; (d) TSHB; (e) GH1.

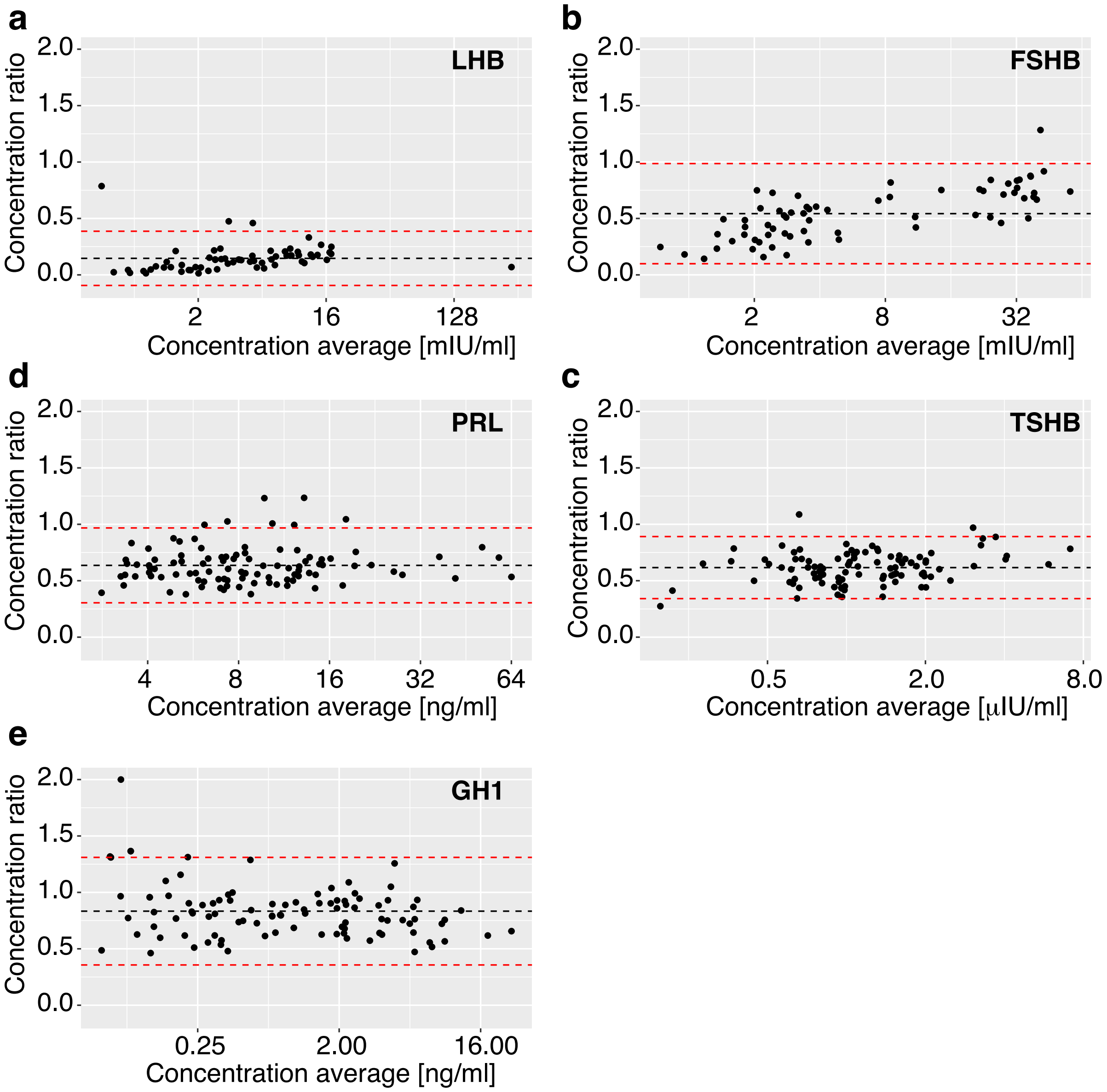

.

Figure S8. Agee association of protein levels. The boxplots include three age groups: women < 45 years of age, 45-55, and >55 years of age for LHB (top row) and FSHB (bottom row). Left plots show qDBS from multiplex assays while right plots show plasma analyzed at the clinical chemistry laboratory. Wilcoxon test applied to the hormone concentrations of the age groups in women is displayed: * $p < 0.05$; ** $p < 0.01$; *** $p < 0.001$; **** $p < 0.0001$.

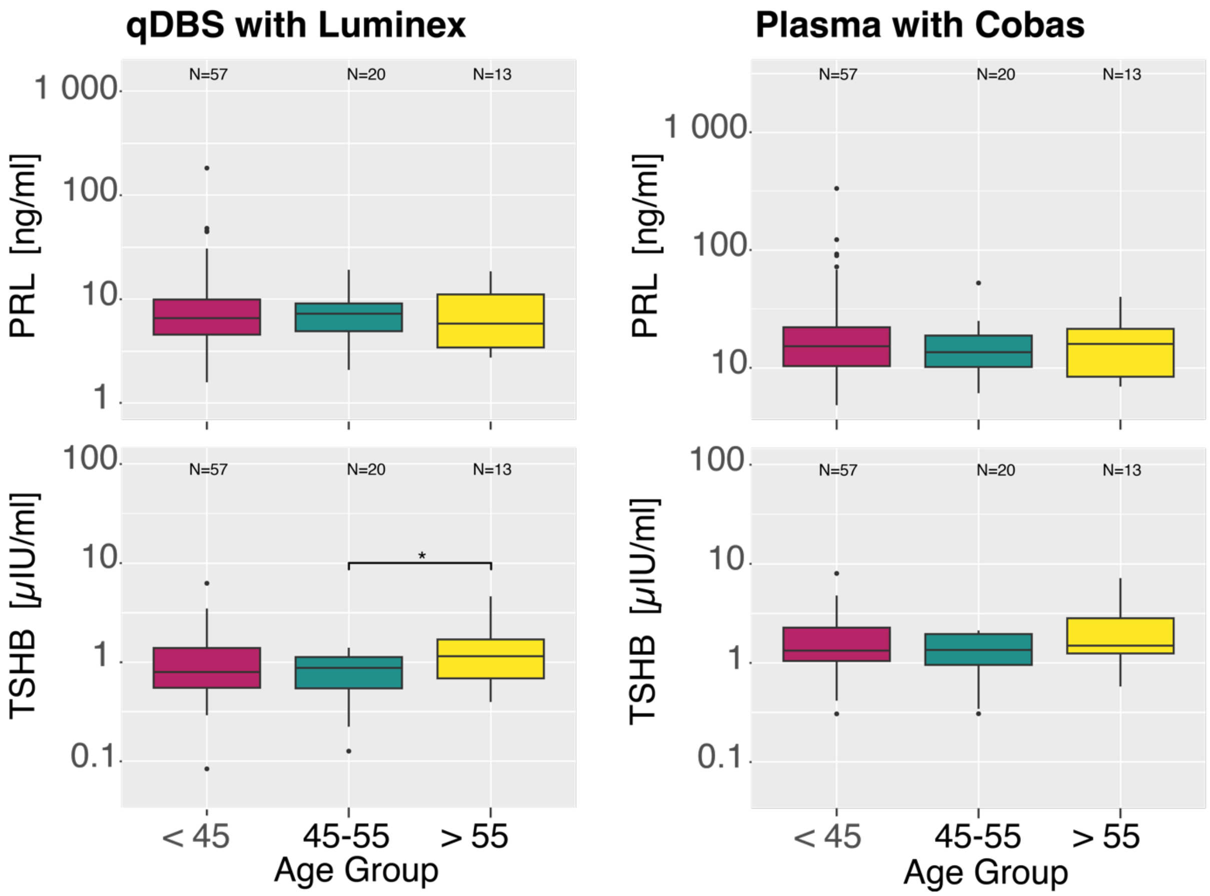

Figure S9. RNA expression level of proteins. RNA expression levels shown as normalized TPN (nTPM) were obtained from the Human Protein Atlas portal. See URL v23.proteinatlas.org/about/assays+annotation#normalization_rna for details.

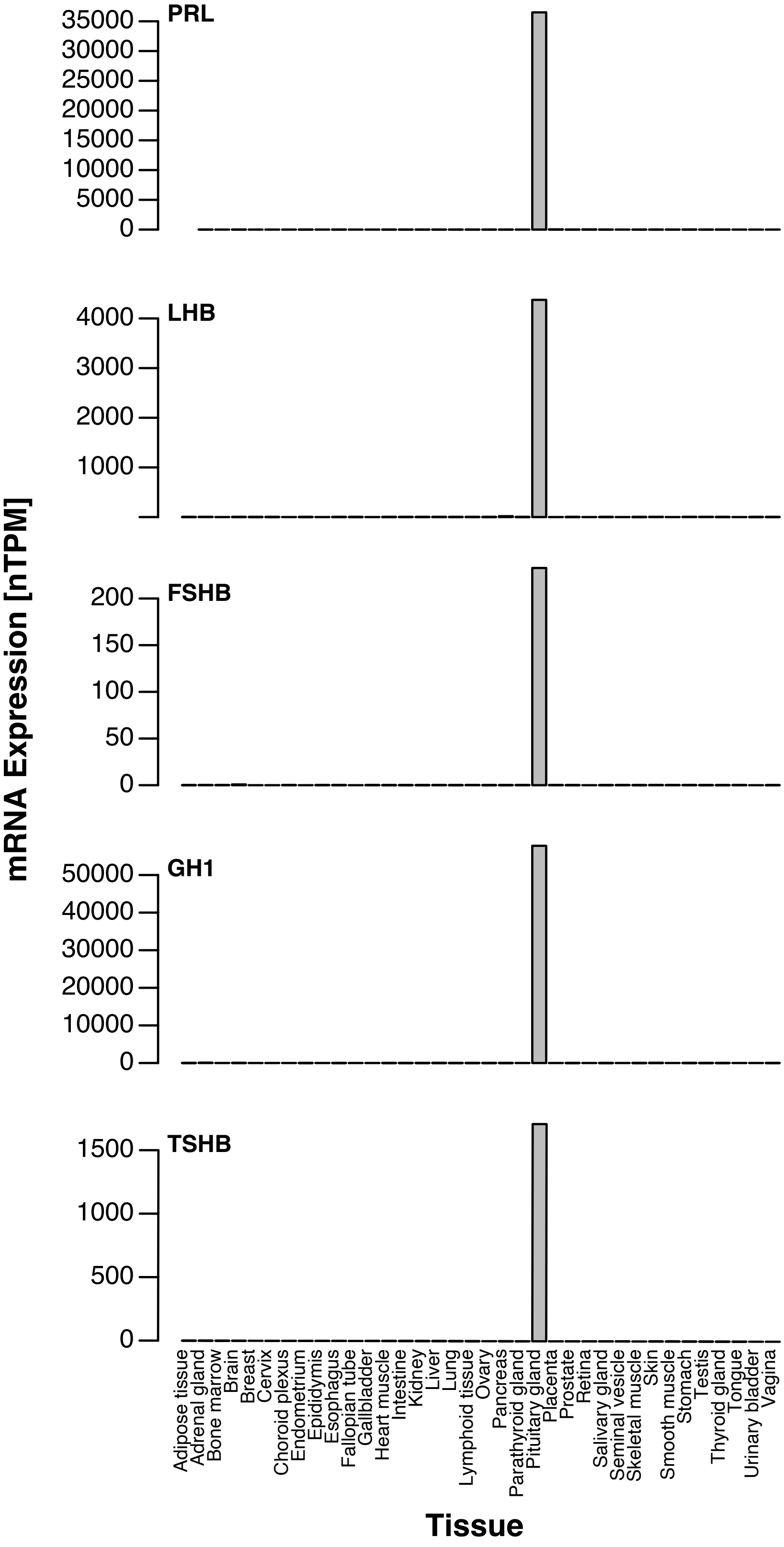

Table S1. Detection metrics for Roche Cobas. Four metrics: Interval, limit of blank (LOB), lower limit of detection (LLOQ), and lower limit of quantification (LLOQ) with CV ≤20%, as specified by the supplier.

	Interval	LOB	LLOD	LLOQ
**FSHB [mU/ml]**	0.3–200	0.1	0.3	1.0
**TSHB [µIU/mL]**	0.005-100	0.0025	0.005	0.005
**PRL [µIU/mL]**	2–10000	1.0	2.0	20
**LHB [mIU/mL]**	0.3–200	0.1	0.3	1.0

Table S2. Correlation analysis for plasma and qDBS (Luminex). A linear regression (Pearson) was used to determine the correlation between protein concentrations in plasma against qDBS from multiplex immunoassay (Luminex).

	LHB	FSHB	PRL	TSHB	GH1
**R**	0.88	0.97	0.93	0.94	0.99
**r^2**	0.77	0.94	0.87	0.89	0.97
**p-value**	$<2 \times 10^{-16}$	$<2 \times 10^{-16}$	$<2 \times 10^{-16}$	$<2 \times 10^{-16}$	$<2 \times 10^{-16}$
**slope**	0.61	0.74	0.89	0.84	1
**intercept**	0.91	0.46	0.3	0.21	0.1

Table S3. LODs of multiplex immunoassays. Limit of detection as specified by the supplier and the average of the plate-specific metrics (N=6).

LOD	LHB [mIU/ml]	FSHB [mIU/ml]	PRL [ng/ml]	TSHB [µIU/ml]	GH1 [ng/ml]
**Lab**	0.038	0.039	0.013	0.008	0.007
**Supplier**	0.044	0.046	0.008	0.004	0.008

Table S4. Correlation analysis for Luminex and Cobas (plasma samples). Pearson correlation (r, r^2, p-value) and linear regression analysis (slope, intercept) were used to determine the relationships between protein concentrations in plasma from multiplex immunoassay (Luminex) and clinical analysis (Cobas).

	LHB	FSHB	PRL	TSHB
**r**	0.95	0.99	0.94	0.87
**r^2**	0.90	0.98	0.89	0.75
**P-value**	$<2 \times 10^{-16}$	$<2 \times 10^{-16}$	$<2 \times 10^{-16}$	$<2 \times 10^{-16}$
**Slope (b)**	1.1	1.1	0.95	0.86
**Intercept**	-0.61	-0.42	-0.077	-0.037

Table S5. Correlation analysis for qDBS (Luminex) and plasma (Cobas). Pearson correlation (r, r^2, p-value) and linear regression analysis (slope, intercept) were used to determine the relationships between protein concentrations in multiplex immunoassay (Luminex) against plasma from clinical analysis (Cobas).

	**LHB**	**FSHB**	**PRL**	**TSHB**
**r**	0.88	0.97	0.94	0.76
**r^2**	0.77	0.95	0.87	0.58
**P-value**	$<2 \times 10^{-16}$	$<2 \times 10^{-16}$	$<2 \times 10^{-16}$	$<2 \times 10^{-16}$
**Slope (b)**	1.5	1.3	0.99	0.84
**Intercept**	-2	-0.96	-0.33	-0.25

Table S6. Multi-age group comparison. Kruskal-Wallis tests were used to determine nominal p-values for the differences in hormone concentrations across the age groups in the three data sets.

	**LHB**	**FSHB**	**PRL**	**TSHB**	**GH1**
**EDTA plasma (Luminex)**	7×10^{-7}	5×10^{-10}	0.6	0.3	0.5
**qDBS (Luminex)**	5×10^{-3}	1×10^{-9}	1	0.2	0.8
**EDTA plasma (Cobas)**	2×10^{-8}	4×10^{-10}	0.7	0.2	N/A

Table S7. Age association. A linear regression was used to determine the nominal p-values for the differences in hormone concentrations across the age groups in the three data sets.

	**LHB**	**FSHB**	**PRL**	**TSHB**	**GH1**
**Plasma (Luminex)**	0.9	2×10^{-14}	0.3	0.7	0.009
**qDBS (Luminex)**	0.2	3×10^{-12}	0.5	0.5	0.02
**Plasma (Cobas)**	0.01	1×10^{-14}	0.4	0.8	N/A
